# Mental Representations of Weekdays

**DOI:** 10.1371/journal.pone.0134555

**Published:** 2015-08-19

**Authors:** David A. Ellis, Richard Wiseman, Rob Jenkins

**Affiliations:** 1 School of Psychology, University of Lincoln, Lincoln, United Kingdom; 2 School of Psychology, University of Hertfordshire, Hatfield, United Kingdom; 3 Department of Psychology, University of York, York, United Kingdom; Birkbeck, University of London, UNITED KINGDOM

## Abstract

Keeping social appointments involves keeping track of what day it is. In practice, mismatches between apparent day and actual day are common. For example, a person might think the current day is Wednesday when in fact it is Thursday. Here we show that such mismatches are highly systematic, and can be traced to specific properties of their mental representations. In Study 1, mismatches between apparent day and actual day occurred more frequently on midweek days (Tuesday, Wednesday, and Thursday) than on other days, and were mainly due to intrusions from immediately neighboring days. In Study 2, reaction times to report the current day were fastest on Monday and Friday, and slowest midweek. In Study 3, participants generated fewer semantic associations for “Tuesday”, “Wednesday” and “Thursday” than for other weekday names. Similarly, Google searches found fewer occurrences of midweek days in webpages and books. Analysis of affective norms revealed that participants’ associations were strongly negative for Monday, strongly positive for Friday, and graded over the intervening days. Midweek days are confusable because their mental representations are sparse and similar. Mondays and Fridays are less confusable because their mental representations are rich and distinctive, forming two extremes along a continuum of change.

## Introduction

To keep appointments and to honor social commitments, it is helpful to know what day it is. In practice, this requirement is not always met. The current studies were motivated by the informal observation that weekday confusions are common in daily life. For example, a person might think that it is Wednesday when in fact it is Thursday. In this paper we argue that confusions between weekdays are highly systematic, and that their distribution reveals much about mental representations of weekdays and their structure. As well as their theoretical interest, the findings have implications for decision making in a wide range of applied settings.

Errors in perception and behavior have often been used to infer underlying cognitive processes, and errors in time perception are no exception (see Gregory, 2009, for an interesting history) [1, 2]. On the millisecond scale, psychophysical studies have revealed distortions of subjective duration and event order associated with changes in physiological or attentional state [3–5]. At longer timescales, biopsychological studies have examined correlates of daily, monthly, and yearly cycles [6–12]. These natural time cycles are derived from the movements of celestial bodies—the rotation of the Earth on its axis, the orbit of the Moon around the Earth, and the orbit of the Earth around the Sun, respectively. Given the stability of these cycles on the evolutionary timescale, it may not be surprising that many biological processes track them [7]. However, much of modern human social activity is organized around the seven-day week [13, 14]. Unlike other calendar cycles, the seven-day week is an entirely human construct. This makes it an interesting cycle from a theoretical perspective. If weekday has psychological consequences, they must be driven by social factors such as cultural norms, rather than by physical or biological factors such as evolutionary pressure.

In fact, a small body of research has identified regularities between weekday and behavior, and also between weekday and mood. Across studies on these topics, two main patterns are emphasized. One is the so-called Blue Monday effect. In a wide range of situations and measures, outcomes are especially negative on Mondays. Many of these situations are non-trivial, as they pertain to health and economic matters. For example, heart attack risk is higher, suicide rate is higher, reported mood is lower, and stock returns are lower [15–20]. Especially positive outcomes on Fridays have also been reported, but with less consistency [21, 22]. This pattern suggests that, at least in terms of mood, Mondays (and possibly Fridays) may be qualitatively different from the other days of the week, which are themselves relatively undifferentiated.

A second pattern emphasizes gradual change from negative to positive through the week [23, 24]. For example, Ellis & Jenkins (2012) found that medical appointments on Mondays were much more likely to be missed than appointments on Fridays. Critically, the rate of missed appointments declined monotonically over the intervening days. This pattern suggests that, rather than being qualitatively different, Monday and Friday may be two extremes along a continuum of change.

The observation of weekday effects across domains as disparate as healthcare and economics hints at a potentially deep connection between the weekly cycle, core cognition, and downstream behavioral outcomes. Such a connection could have far-reaching implications, for the simple reason that so many of us are in phase with respect to the weekly cycle—when it’s Monday, it’s Monday for all of us. With such widespread synchrony, any psychological consequences of weekday should be highly correlated across individuals, and should tend to sum rather than to cancel out. The implication is that even small effects could scale up to systematic biases at the population level [24]. For example, it is already well established that affective state can influence decision-making [25]. If different days of the week are associated with different affective profiles, the outcome of a decision could depend on the day on which it is taken.

Our aim in this paper is to illuminate possible links between the weekly cycle and basic cognition by characterizing mental representations of weekdays. An important limitation of previous studies is that they have typically looked at a single outcome measure in isolation, such as reported mood or attendance rate. That is, they have analyzed similarity of weekdays along a single psychological dimension rather than their overall similarity, as indexed by confusability. Confusability is a particularly useful measure, as it requires no assumptions about which dimensions of similarity are most important [26–29]. However, the only previous studies to look at confusability of weekdays have done so from the perspective of serial order memory [30, 31]. On the face of it, serial order memory effects seem an unlikely explanation for weekly fluctuations in measures such as suicide and heart attack rates. For this reason, we take a very different approach that emphasizes the semantic and affective *character* of weekdays, rather than their *order*. The eventual aim is to understand behavioral effects of weekday for which serial order memory is not an easy explanation.

We began by tracking the prevalence of weekday confusability over the seven-day cycle (Study 1). To trace the resulting pattern to mental representations of weekdays, we then compared retrieval speed for the current day on different days of the week (Study 2). Finally, to identify determinants of weekday confusability at the level of mental representation, we analyzed semantic associations for each weekday, specifically their numerosity and affective valence (Study 3). Together, these studies show that confusability of weekdays is highly systematic, and imply that temporal cycles can shape cognition even when they are socially constructed.

### Ethics Statement

The University of Glasgow, College of Science and Engineering Ethics Committee approved all research. Participants were informed about procedures in detail and provided written informed consent.

## Study 1

In setting out this study, it is useful to distinguish between *actual* day (i.e., the current weekday according to the calendar) and *apparent* day (i.e., the weekday that the current day feels like, according to the respondent). This distinction allows us to test for mismatches between actual day and apparent day.

Our general approach to data collection was to elicit apparent weekday from respondents on each day of the week, so that data was collected over the whole seven-day cycle. In this way, we sought to establish whether mismatches are distributed evenly across the week, or whether they cluster in reliable patterns. Given that the working week (Monday to Friday) and the weekend (Saturday and Sunday) often involve rather contrasting routines, we expected that weekends might provide an especially salient marker in the weekly cycle. If so, then Mondays and Fridays should be relatively distinctive, because they begin and end the working week. In contrast, Tuesdays, Wednesdays, and Thursdays should be less distinctive, as none of them adjoins the weekend.

Our main interest was in the following patterns. First, the probability that the apparent day matches the actual day may differ through the week. For example, Fridays might consistently feel like Fridays, whereas Wednesdays might frequently feel like other weekdays. Although previous research has examined weekday confusion in the context of memory for past events [30, 31], none has addressed the more basic question of situating oneself in the seven-day cycle. Second, apparent day might be attracted more strongly by some days of the week than others. For example, apparent day might lag or lead actual day by a regular interval (e.g. one day). Alternatively, any day might be equally likely to be mistaken for any other. Third, mismatch rate may be determined in part by transitions between the weekend and the working week. To isolate the role of such transitions in regulating mental representations of weekday, we compared distributions of mismatches for a Normal week and a Bank Holiday week, in which the Monday was a public holiday.

### Method

#### Design

In this study, we assessed apparent day as a function of actual day, in order to track the correspondence between the two over the seven-day cycle. Apparent day was assessed via 7AFC response. Actual day was manipulated between subjects by collecting data on every day of the week.

To enable recruitment of a broad cross-section of participants, and to facilitate data collection of the weekend, the study was conducted online using Survey Monkey (www.surveymonkey.com). Anonymous IP logging was used to filter out multiple responses from the same computer, and to tag responses with time zone and nation.

To dissociate effects of weekend/working week transition from effects of calendar weekday, we ran the study in two consecutive weeks. Week 1 was a Normal working week in every country from which responses were received. Week 2 was a Bank Holiday week in the UK and some other countries, meaning that the Monday of that week was a public holiday rather than being a working day. Comparing responses in these two weeks allowed us to examine any effects on apparent day due to delaying the onset of the working week by one day.

#### Participants

A total of 1115 respondents contributed data in two weeks of May 2009. This was a convenience sample recruited via Facebook, Twitter, and various blogs to ensure that data was collected evenly across each week (80 responses per day on average).

In Week 1 (Normal week), 502 respondents took part [301 female, 201 male; modal age bands 21–30 (41%), 31–40 (26%); modal locations UK (59%), Other Europe (24%), North America (14%)]. In Week 2 (Bank Holiday week), 613 respondents took part [248 female, 365 male; modal age bands 21–30 (37%), 31–40 (24%); modal locations UK (66%), Other Europe (8%), North America (21%)].

#### Procedure

The procedure began with presentation of the following prompt for apparent weekday, which remained onscreen until response:

“People sometimes have the feeling that they are on the wrong day of the week. For example, it might ‘feel like’ a Friday when it is in fact Wednesday. What day of the week does today feel like to you?”

Participants indicated their response by selecting one of the seven weekdays from a drop down menu (7AFC). They were then asked to select their gender, age band, and current location (country only).

### Results

#### Coding

Responses included a GMT (Greenwich Meantime) field specifying the time of submission. For each response, this time field was adjusted for time zone to ensure that actual day was correctly coded according to the respondent’s local frame of reference. Responses were coded as Bank Holiday Week if the Monday of that week was a public holiday in the respondent’s country, and Normal Week if it was not.

#### Analysis

**Fig 1:**
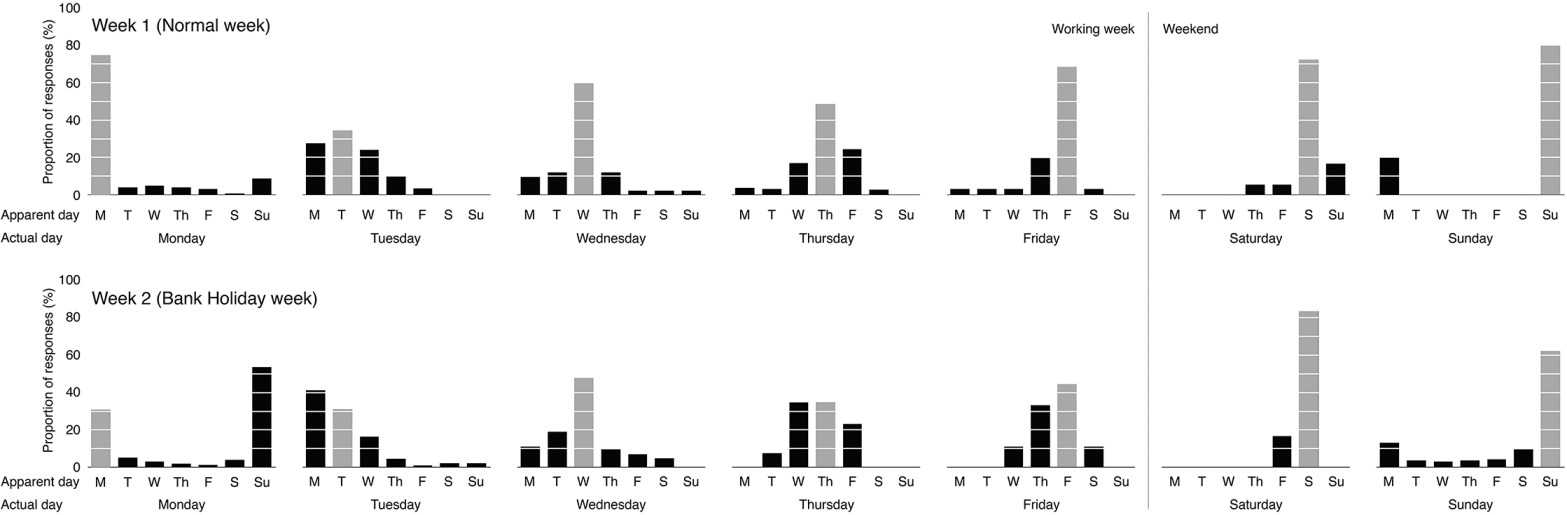
Proportions (%) of apparent day responses for each actual day, shown separately for Normal Week (top row) and Bank Holiday Week (bottom row). Grey bars denote matches between apparent day and actual day. Black bars denote mismatches.

Fig 1 shows the proportion of apparent day responses as a function of actual day, separately for Week 1 (Normal week) and Week 2 (Bank Holiday week). Several informative patterns are evident. We consider each data set in turn before addressing differences between them.

#### Week 1 (Normal week)

Mismatches between apparent day and actual day were surprisingly common, accounting for 37.5% of responses overall. As can be seen from Fig 1, these mismatches were not distributed evenly through the week, but instead formed definite clusters. Mismatches were relatively frequent on Tuesdays (65.6%), Wednesdays (40.5%), and Thursdays (51.4%), and relatively infrequent on Mondays (25.4%), Fridays (31.8%), and weekend days (Saturdays, 27.8%; Sundays, 20.0%). Chi-square analysis of frequency data confirmed that mismatches were more frequent for midweek days (Tuesday, Wednesday, and Thursday) than for working week/weekend transitions (Monday, Friday, Saturday, and Sunday) [*Χ* ^2^ (1, N = 502) = 28.7, *p* < 0.001]. Actual day (Day 0) was often reported to feel like either the immediately preceding day (Day -1; 31.6% of mismatches), or the immediately following day (Day +1; 42.7%). Most of the remaining mismatches involved Day -2 (8.9%) or Day +2 (6.6%). Mismatches involving Day -3 or Day +3 were rare (5.0% and 5.2% respectively).

#### Week 2 (Bank Holiday week)

In the Bank Holiday week, mismatches between apparent day and actual day outnumbered matches, accounting for 52.2% of responses overall. Mismatch rate was high throughout the working week (Mondays, 69.0%; Tuesdays, 68.8%; Wednesdays, 52.4%; Thursdays, 65.4%; Fridays 55.6%), and did not fall sharply until the weekend (Saturdays, 16.7%, Sundays 37.8%). Chi-square analysis found no significant difference in the frequency of mismatches midweek (Tuesday, Wednesday, and Thursday) and at working week/weekend transitions (Monday, Friday, Saturday, and Sunday) [*X* ^2^ (1, N = 613) = 1.43, *p* > 0.2]. The majority of these mismatches were confusions between the actual day with the immediately preceding day (Day -1, 58.8%). In particular, Monday was reported to feel like Sunday, and Tuesday was reported to feel like Monday. Confusions with the immediately following day were much less common (Day +1, 20.0%). Most of the remaining mismatches involved Day -2 (10.5%) or Day +2 (4.9%). As in the Normal Week, mismatches involving Day -3 or Day +3 were rare (5.0% and 5.2% respectively).

#### Comparison of Normal Week and Bank Holiday Week

Mismatches between apparent day and actual day were more frequent in the Bank Holiday Week (52.2%) than in the Normal Week (37.5%) [*X* ^2^ (1, N = 1115) = 37.1, *p* < 0.001]. Moreover, the distribution of these mismatches was strikingly different. For the Normal Week, mismatches were clustered midweek (Tuesday, Wednesday, Thursday), and involved confusion with preceding days and following days in roughly equal proportion (e.g. Day -1 or Day +1). In contrast, for the Bank Holiday Week, mismatches were common throughout the working week (Monday to Friday), and were much more likely to involve confusion with the preceding days (Day -1) than with the following day (Day +1). Thus, apparent day lagged actual day in the Bank Holiday Week, but not in the Normal Week [*X* ^2^ (1, N = 852) = 71.1, *p* < 0.001].

### Discussion

Mismatches between apparent day and actual day were surprisingly common in this study. In more than a third of responses, participants reported that the current day felt like a different day. Importantly, these mismatches were not randomly distributed through the week, but instead followed a systematic pattern. First, mismatches were reported more frequently on midweek days (Tuesday, Wednesday, and Thursday) than on other days (Monday, Friday, Saturday, Sunday), perhaps reflecting a midweek dip in psychological salience. Second, mismatches were mainly intrusions from neighboring days, rather than by more distant days. This pattern was particularly clear for the midweek days, consistent with graded similarity of their mental representations. Third, Bank Holiday Monday induced a ‘one-back’ effect, such that Monday felt like Sunday, Tuesday felt like Monday, and so on—an effect that persisted until the weekend. This Bank Holiday effect implies that apparent weekday is not determined solely by the seven-day period of the weekly cycle: transitions between working week and weekend also play a role.

One way to think about these transitions is in terms of category boundaries [32]. If the week is broken into two categories—working week and weekend [30]—then mismatches should occur more frequently within categories than between categories. In order to accommodate anomalies such as Bank Holiday Monday, any such categories would have to be flexible, perhaps being determined by semantic or behavioral associations rather than by fixed verbal labels. We return to the role of weekday associations in Study 3.

The relatively high frequency of mismatches on midweek days suggests that mental representations of those weekdays may be relatively indistinct. However, the mismatch measure specifically emphasized which weekday the current day *feels like* rather than which weekday the current day *is*. In the next study we address the latter question directly using a more objective lab-based measure.

## Study 2

To test whether midweek days are less salient than other days, we compared ease of access for different weekdays in a reaction time task. Based on the pattern of mismatches seen in Study 1, we predicted that it would be relatively hard to retrieve the current day on Tuesdays, Wednesdays, and Thursdays (resulting in slower response latencies), and relatively easy to retrieve the current day on Mondays and Fridays (resulting in faster response latencies). That is, differentiating between less distinct mental representations should take longer.

### Method

#### Design and apparatus

We used a simple one-shot production task in which participants were required to state the current day. In this task, retrieval was induced by asking participants what day it was, and ease of retrieval was operationalized as reaction time. The spoken retrieval cue (“Can you tell me what day of the week it is today?”) was pre-recorded to standardize presentation, and participants’ verbal responses were recorded for offline reaction time analysis.

We manipulated weekday between subjects by collecting data on each day of the working week (Monday to Friday). Weekend days (Saturday and Sunday) were omitted due to constraints on recruiting participants at the weekend.

#### Participants

Sixty-five University of Glasgow undergraduates (47 female, 18 male; mean age 20 years), who were naïve to the aims of the study, completed the task in exchange for a small payment. Thirteen participants were recruited to each day (Monday to Friday). All were native English speakers, and none had taken part in Study 1.

#### Procedure

Individual participants were informed that a pre-recorded question would be played from the computer, and were asked to speak the answer as quickly as possible. After confirming that the task was clear, the participant triggered the pre-recorded question (“Can you tell me what day of the week it is today?”) via keypress.

### Results

**Fig 2:**
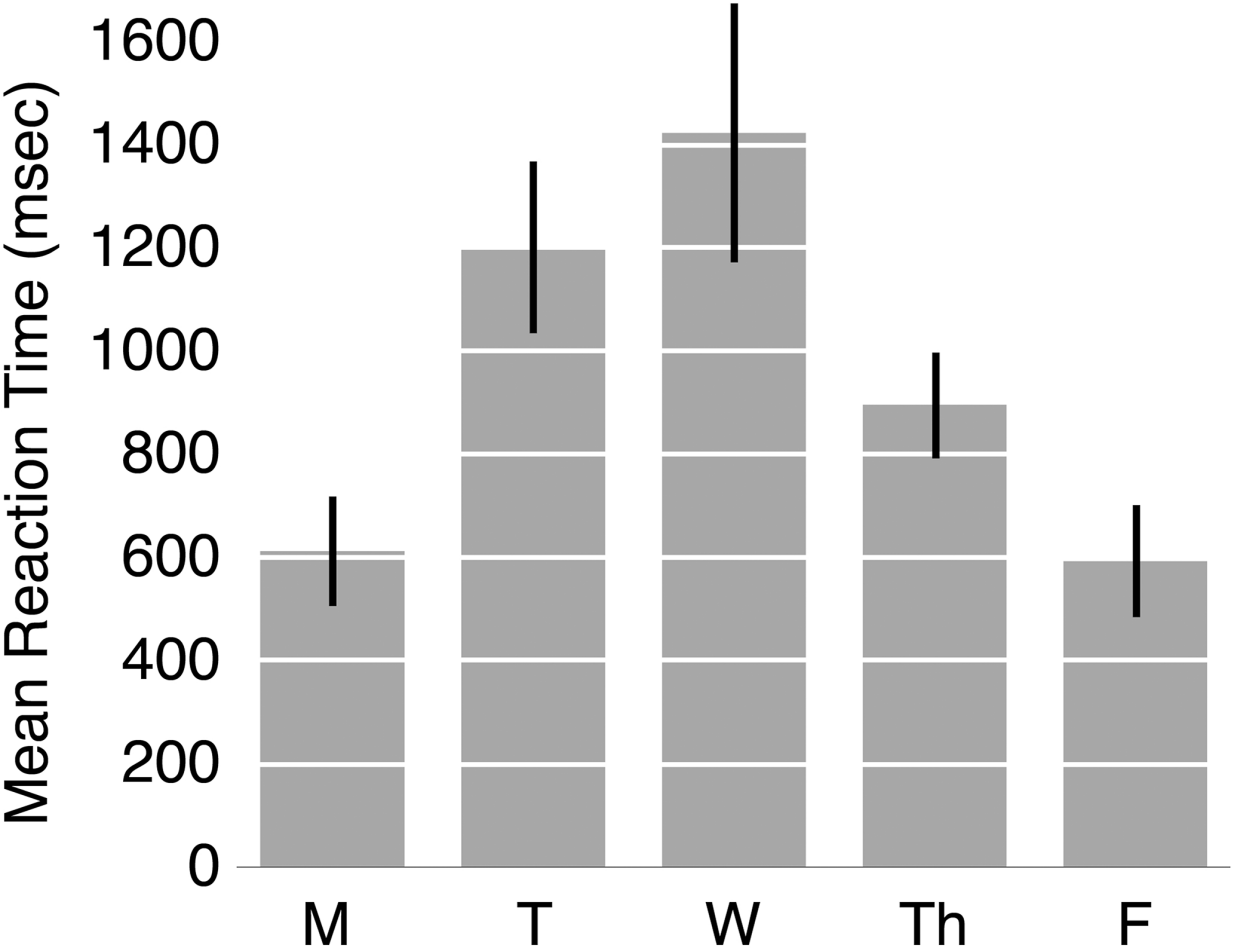
Mean correct reaction times (msec) as a function of weekday for the production task in Study 2. Error bars show SEM.

Reaction times were measured from the offset of the word ‘today’ in the pre-recorded cue to the onset of the participant’s response. Data from two participants who could not recall what day it was (one on a Wednesday and one on a Thursday), were excluded from the analysis. Fig 2 shows mean reaction times for correct weekday responses.

As can be seen from Fig 2, responses were fastest for Monday and Friday, slowest for Wednesday, and intermediate for Tuesday and Thursday. One-way between-subjects ANOVA revealed a significant main effect of weekday on response time [*F*(4, 60) = 5.38, *p* < 0.001, *η* ^*2*^_*p*_ = 0.264]. Pairwise comparisons (Tukey’s HSD) are summarized in Table 1.

**Table 1:** Pairwise comparisons (Tukey’s HSD) between weekdays in Study 2. Cells contain absolute differences in mean reaction times (sec).

	M	T	W	Th	F
Monday	0	0.59	0.81**	0.28	0.02
Tuesday	0.59	0	0.22	0.31	0.61
Wednesday	0.81**	0.22	0	0.53	0.83**
Thursday	0.28	0.31	0.53	0	0.30
Friday	0.02	0.61	0.83**	0.30	0

* *p* < 0.05

** *p* < 0.01.

As Table 1 shows, significant differences were found between Monday (612 msec) and Wednesday (1422 msec), and between Friday (593 msec) and Wednesday. No other comparisons were significant.

### Discussion

As expected from the results of Study 1, the time required to state the current day changed substantially across the working week. Indeed, responses were twice as fast on Monday and Friday as on Wednesday, with intermediate response times for Tuesday and Wednesday, forming a quadratic shaped function over the week. Evidently it was easier for participants to retrieve the current day on Monday and Friday, and harder to do so midweek. Although no categorical errors (i.e. stating an incorrect day) arose in this task, we note that two participants were unable to report the current day, and that both of these participants were both tested midweek (Wednesday and Thursday).

Converging evidence from survey data (Study 1) and experimental data (Study 2) points to reduced psychological salience for midweek days compared with other days. The observed quadratic shaped function is reminiscent of the classic sequence memory phenomenon where items in the middle of a sequence are less well remembered than items at the beginning and the end [33]. A number of computational models have been proposed to account for such effects in sequence memory [31, 34, 35]. But the weekly cycle differs from typical sequence memory materials in several important ways. Unlike other sequences (e.g. shopping lists), the weekly cycle is repeated invariantly from birth, and is used worldwide to organize events and activities [13, 14]. The stability of this cycle over the lifetime, coupled with constant reminders of the current phase, potentially result in each day of the week acquiring its own *character*. In the final study, we test this possibility directly by combining behavioral and informatic measures to investigate semantic and affective associations with each day of the week.

## Study 3

The main aim of this study was to characterize mental representations of weekdays by analyzing their semantic associations. Participants were asked to list associations for each weekday name in the context of a free association task. We first analyzed the number of associations for each day—a measure of representational richness or degree of elaboration [36]. To establish the generality of our lab-based findings, we conducted a complementary informatics analysis based on millions of webpages and books. We also analyzed the affective profile of participants’ weekday associations, using Affective Norms for English Words (ANEW) [37]. Given that midweek days seem to be especially confusable, we predicted that Tuesday, Wednesday, and Thursday would elicit relatively few associations compared with other weekdays. We also anticipated contrasting affective valence for Monday associations (negative) and Friday associations (positive) [21, 38].

### Method

#### Design

The purpose of the study was to collect as many or as few associations for each weekday as occurred to our participants, and to examine the emotional profile of these associations. Participants were provided with response sheets consisting of seven empty columns headed by the names of the seven weekdays Monday to Sunday. To circumvent any potential order effects, different participants received these weekday cues in different orders. For each participant, we recorded the total number of associations generated for each weekday. Each association was then scored using the ANEW system [37]. In this system, words are rated on three bipolar dimensions—*Pleasure* (Unhappy–Happy), *Arousal* (Calm–Excited), and *Dominance* (Controlled–In control). Inter-item similarity can then be expressed as linear distance in a three-dimensional affective space.

#### Participants

Sixty undergraduate volunteers from the University of Glasgow (46 female, 14 male; mean age 19 years) completed the word association task in exchange for course credit. None had taken part in Study 1 or Study 2.

#### Procedure

Participants were asked to write down any words that they associated with each weekday, using the form provided. To ensure that the task was as unconstrained as possible, no additional instruction was provided, and no time limit was imposed.

### Results

#### Number of associations

**Fig 3:**
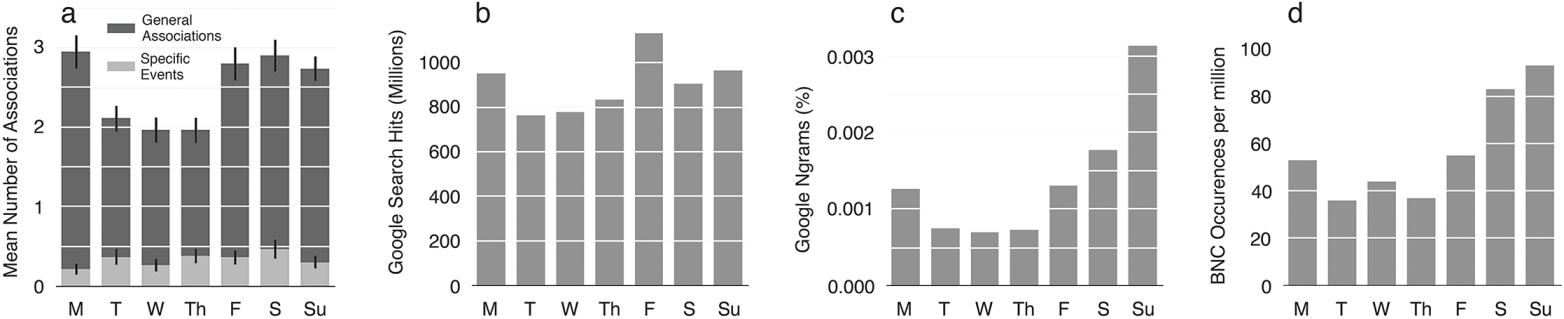
(a) Mean number of associations generated for each weekday in Study 3. General Associations are shown in dark grey, Specific Events in light grey. Error bars show SEM. (b) Number of hits returned by Google Search for each of the search terms “Monday” to “Sunday”. (c) Google Ngram search results. The y-axis shows the percentages of weekday words in the corpus. See main text for details. (d) Word frequencies from the British National Corpus[36].

As our main concern was the affective profile of each weekday, we separated associations that referred to *specific events* (e.g. “Dental appointment”, “Kate’s birthday”) from *general associations* consisting of adjectival descriptors or generic activities (e.g. “fun”, “family”). Fig 3A summarizes the number of associations of each type generated for each day of the week.

As can be seen from Fig 3A, the profile for Specific Events was relatively flat. One-way analysis of variance (ANOVA) found no significant difference in the number of specific events associated with each weekday [*F*(6, 354) = 1.24, *p* = 0.283, *η* ^*2*^_*p*_ = 0.021]. In contrast, the profile for General Associations was markedly scooped, with fewer associations generated for Tuesday, Wednesday, and Thursday, compared with other weekdays. One-way analysis of variance (ANOVA) revealed a significant main effect of weekday on the number of General Associations produced [*F*(6, 354) = 9.98, *p* < 0.001, *η* ^*2*^_*p*_ = 0.145], and on the total number of associations (General Associations plus Specific Events) [*F*(6, 354) = 9.85, *p* < .0001, *η* ^*2*^_*p*_ = 0.143]. Pairwise comparisons (with Bonferroni correction) are summarized in Table 2.

**Table 2:** Pairwise comparisons (with Bonferroni adjustment) between weekdays in Study 3. Cells contain absolute differences in the number of associations generated for each day.

	M	T	W	Th	F	S	Su
Monday	0	0.83**	0.98**	0.98**	0.15	0.05	0.22
Tuesday	0.83**	0	0.15	0.15	0.68*	0.78**	0.61
Wednesday	0.98**	0.15	0	0	0.83**	0.93**	0.76**
Thursday	0.98**	0.15	0	0	0.83**	0.93**	0.76**
Friday	0.15	0.68*	0.83**	0.83**	0	0.10	0.06
Saturday	0.05	0.78**	0.93**	0.93**	0.10	0	0.16
Sunday	0.22	0.61	0.76**	0.76**	0.06	0.16	0

* *p* < 0.05

** *p* < 0.01.

As Table 2 shows, significant differences emerged between Monday and each of the midweek days (Tuesday, Wednesday, Thursday), between Friday and each of the midweek days, and between Saturday and each of the midweek days. Significant differences were also observed between Sunday and both Wednesday and Thursday. No other comparisons were significant.

#### Informatics

The behavioral finding that midweek days evoked fewer associations than did other days raises the question of why this should be the case. One possibility is that midweek days occur relatively infrequently in natural language, thus providing fewer opportunities for associations to form. To test this possibility, we used Google Search [http://www.google.com] to compare hits for weekday names on the internet, and Google Ngrams [http://books.google.com/ngrams] to compare the frequency of their occurrence in books (>5 million books in total) [39]. The Google Ngrams search was based on the English 2012 corpus, and included English language books published in America between 1958 and 2008 inclusive. Smoothing was set to 50 years to yield a single number for each weekday. Both searches were conducted in August 2013.

Results from these searches are shown in Fig 3B and 3C. Interestingly, both distributions exhibit the scooped profile of the associations data, despite their very different sources. Fig 3D shows word frequencies from the British National Corpus [40]. In all four data sets, the midweek days Tuesday, Wednesday, and Thursday are underrepresented, relative to other weekdays, echoing the quadratic functions for salience seen in Studies 1 and 2.

#### Affective content of weekday associations

**Fig 4:**
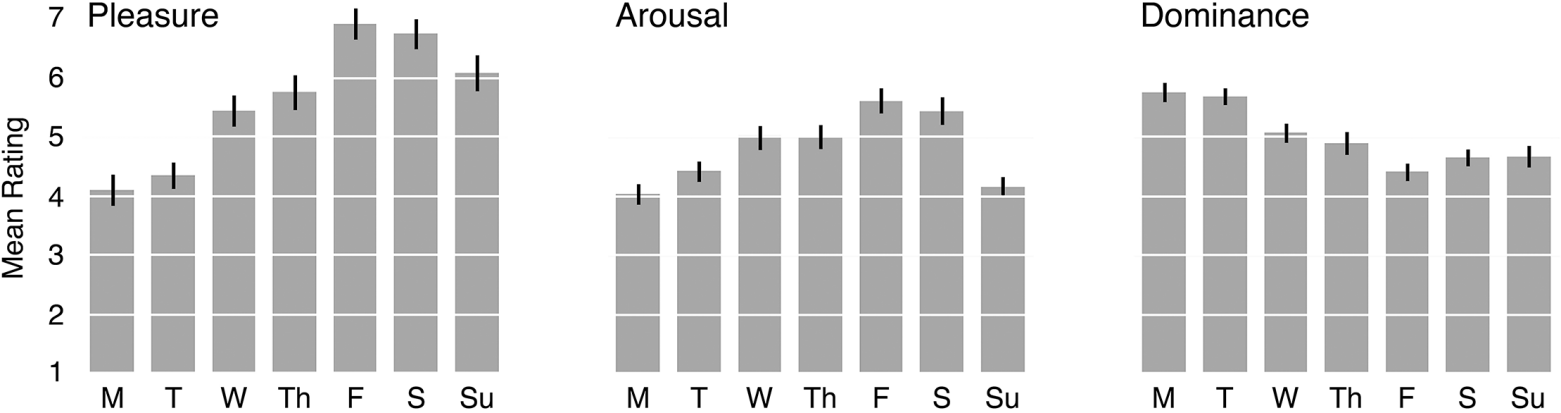
Mean *Pleasure* (left), *Arousal* (centre), and *Dominance* (right) ratings of associations generated for each weekday in Study 3. Error bars show SEM.

To examine the affective content of participants’ weekday associations, we recruited thirty independent raters (12 male, 18 female; mean age 23.1) to score the General Associations using the ANEW system [37]. Each item was scored on three dimensions (*Pleasure*, *Arousal*, and *Dominance*) using a nine-point scale. Mean scores were computed for each of these associations by averaging scores across raters. These means were then pooled by weekday to give overall *Pleasure*, *Arousal*, and *Dominance* ratings for each day of the week (see Fig 4).

In contrast to the quadratic functions seen in our measures of salience, each of the three affective dimensions shows a more linear change over the working week (Monday to Friday). *Pleasure* increases steeply from Monday to Friday before dipping slightly over the weekend. *Arousal* climbs through the week, and then drops sharply on Sunday. *Dominance* begins high and decreases monotonically, with a slight recovery over the weekend. Separate one-way ANOVAs conducted on these ratings revealed significant differences in *Pleasure* [*F*(6, 633) = 28.54, *p* < 0.001, *η* ^*2*^_*p*_ = 0.213], *Arousal* [*F*(6, 633) = 18.69, *p* < 0.001, *η* ^*2*^_*p*_ = 0.150] and *Dominance* [*F*(6, 633) = 18.142, *p* < 0.001, *η* ^*2*^_*p*_ = 0.147], as a function of weekday. Pairwise comparisons are shown in Table 3.

**Table 3:** Pairwise comparisons (Tukey’s HSD) between weekdays for the *Pleasure* (left), *Arousal* (centre), and *Dominance* (right) ratings in Study 3.

	Pleasure							Arousal							Dominance						
	M	T	W	Th	F	S	Su	M	T	W	Th	F	S	Su	M	T	W	Th	F	S	Su
M	0	-	**	**	**	**	**	0	-	**	**	**	**	-	0	-	**	**	**	**	**
T	-	0	*	**	**	**	**	-	0	-	-	**	**	-	-	0	*	**	**	**	**
W	**	*	0	-	**	**	-	**	-	0	-	-	-	**	**	*	0	-	**	-	-
Th	**	**	-	0	**	-	-	**	-	-	0	-	-	**	**	**	-	0	-	-	-
F	**	**	**	**	0	-	*	**	**	-	-	0	-	**	**	**	**	-	0	-	-
S	**	**	**	-	-	0	-	**	**	-	-	-	0	**	**	**	-	-	-	0	-
Su	**	**	-	-	*	-	0	-	-	**	**	**	**	0	**	**	-	-	-	-	0

* *p* < 0.05

** *p* < 0.01.

#### Affective similarity of weekdays

To determine which days are most and least similar in emotional profile, we next computed linear distances between them in affective space.

In this analysis, a three-dimensional space is constructed from the *Pleasure*, *Arousal*, and *Dominance* dimensions of the ANEW system, and the mean ratings of the weekdays give their coefficients on these dimensions. Each weekday thus occupies a single point in the affective space, and the distance between two points is the root sum of squared differences on each dimension.

**Fig 5:**
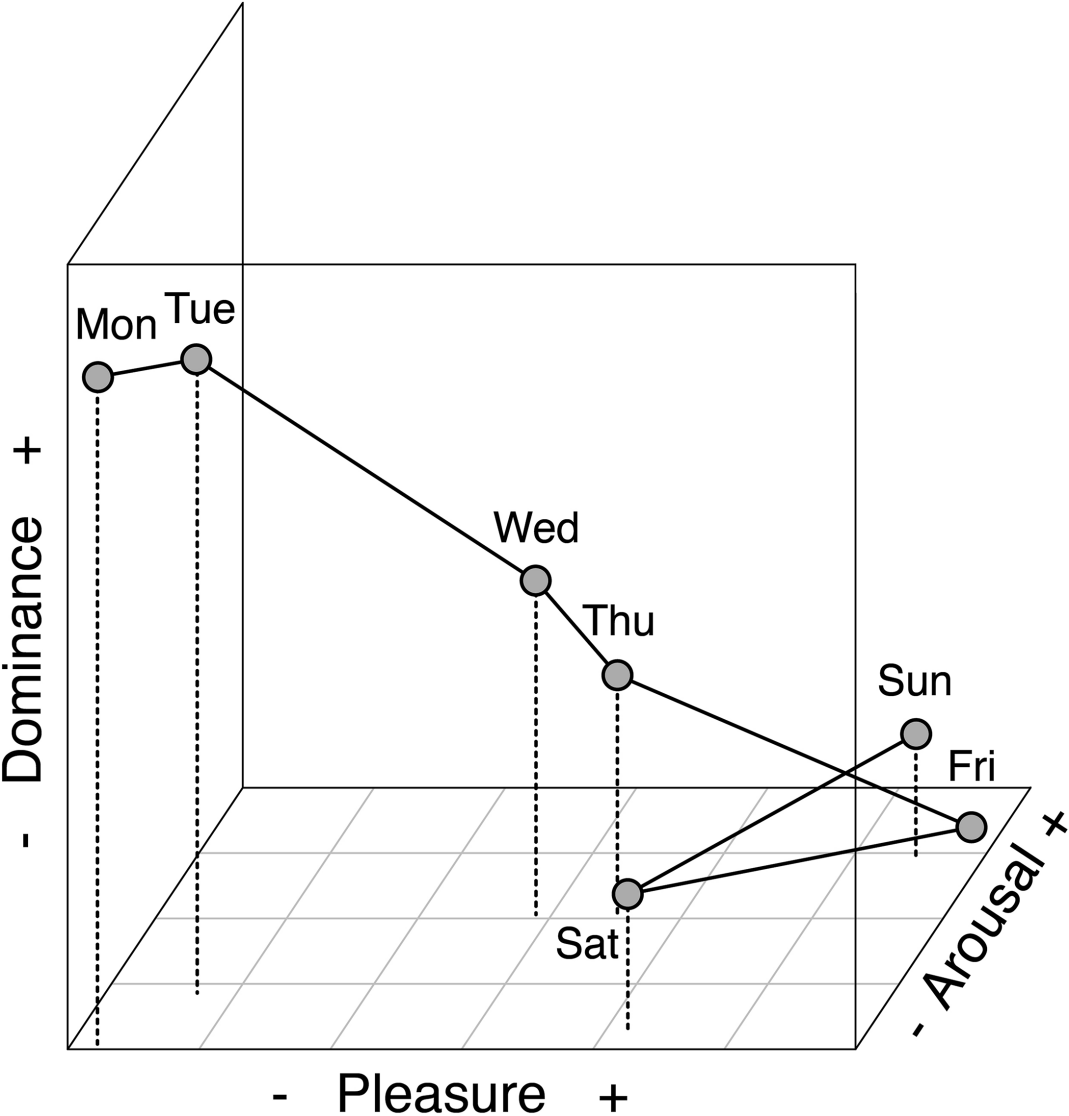
Linear distances between weekdays in affective space, computed from *Pleasure*, *Arousal*, and *Dominance* ratings in Study 3 (see main text for details).

Fig 5 summarizes the results of this analysis. As can be seen from the figure, the greatest distance is between Monday and Friday, indicating that these two days differ the most in affective profile. In contrast, the distances between the midweek days—especially Wednesday and Thursday—are relatively short, implying greater similarity [41]. This analysis reveals an important consequence of the graded continuum on each dimension: for each day of the working week (Monday to Friday), adjacent days were always most similar, in terms of their emotional profile.

### Discussion

In a behavioral word association task, participants generated significantly fewer associations for midweek days (Tuesday, Wednesday, Thursday) than for other weekdays. The association effect is unlikely to reflect a dip in busyness, as the number of events associated with each day was statistically flat across the week. Moreover, in separate informatics analyses, we found fewer occurrences of midweek days than other weekdays on the internet (Google Search), in books (Google Ngram Search), and in the British National Corpus [40]. Together, these findings suggest that less is said about midweek days than about other weekdays, leading to relatively sparse networks of association.

Analysis of the emotional content of these weekday associations revealed two important insights. First, the strongest contrast in affect was between Monday (lowest *Pleasure*, lowest *Arousal*, highest *Dominance*) and Friday (highest *Pleasure*, highest *Arousal*, lowest *Dominance*). Second, ratings on all three dimensions varied remarkably smoothly, with an approximately linear function over the working week. Thus, for each working day (Monday to Friday) the days that were affectively most similar were the immediately preceding day (Day -1), and the immediately following day (Day +1). This pattern of affective similarity accords with both the pattern of mismatches seen in Study 1, where apparent day was typically adjacent to actual day, and the pattern of reaction times seen in Study 2, where the fastest responses occurred on Mondays and Fridays.

## General Discussion

We initially set out to investigate a widely experienced cognitive slip in which one day of the week feels like another. We found that mismatches between apparent day and actual day are highly systematic, and can be traced to the similarity of their mental representations. Converging evidence from large online survey data, experimental data, and informatics supports these conclusions. In Study 1, respondents reported mismatches between apparent day and actual day surprisingly often (37.5% of responses in a normal week). Mismatches were more frequent on midweek days than on other days, and mainly involved confusions with the immediately preceding or the immediately following day. A Bank Holiday Monday increased the overall number of mismatches (52.5% of responses), and skewed apparent day heavily towards the preceding day, so that Monday felt like Sunday, Tuesday felt like Monday, and so on. In a speeded production task (Study 2), we found that reaction times to retrieve the current day were fastest on Monday and Friday, slowest on Wednesday, and intermediate for Tuesday and Thursday. Finally, in Study 3, participants generated significantly fewer associations for midweek days (Tuesday, Wednesday, Thursday) than for other weekdays. This midweek dip was not explained by busyness—the number of scheduled events associated with each day. Google Search (website content) and Google Ngram Search (book content) each returned fewer hits for midweek days than for other days of the week, consistent with data from the associations task and data from the British National Corpus[40]. Analysis of the affective profile of these weekday associations (*Pleasure*, *Arousal*, and *Dominance*) revealed that the strongest contrast on each of these dimensions was between Monday and Friday. The loadings on each dimension varied gradually through the week, such that adjacent days were affectively more similar than non-adjacent days. We take from these findings that Monday and Friday are two extremes along a continuum, rather than being qualitatively different from other weekdays.

All of these findings can be understood in terms of mental representations of weekdays, their distinctiveness, and their richness, where *distinctiveness* is determined by the constellation of concepts associated with a given day, and *richness* is determined by the number of associations [42, 43]. On this view, mental representations of Monday and Friday are both rich and distinctive—Monday being rich and affectively negative, and Friday being rich and affectively positive. In contrast, mental representations of midweek days are more sparse and more homogenous—not only do they attract fewer associations, but those associations tend to be affectively neutral. Study 2 corroborates this view, by showing that the current day is more readily accessed on Mondays and Fridays than midweek. Together, these findings explain the pattern of weekday confusions seen in Study 1. First, the days on which mismatches between apparent day and actual day were most frequent (Tuesday, Wednesday, Thursday) are precisely those with sparse networks of associations. Second, the days that most strongly attracted mismatches (i.e. directly adjacent days) are precisely those with the most similar affective profiles.

We noted in Study 1 the quadratic function for weekday salience over the week, and a similar pattern emerged in Studies 2 & 3. This pattern resembles the serial position curves seen in sequence order memory studies, in which items at the beginning and the end of a sequence are better remembered than items in the middle. This resemblance raises the question of whether weekday effects are in any way specific to the weekly cycle, or whether they are merely another instance of a more general phenomenon, albeit one with potentially broad implications for everyday life. A full account of weekday effects would have to accommodate not only affective patterns of the type seen here (Study 3), but also the broad spectrum of weekday effects seen in health and economic settings—including effects on suicide rate, heart attack rate, mood, economic decision making, and other behaviors [15–24]. Whether or not all of these effects can be subsumed under a sequence memory account remains to be seen. An informative test would be whether the affective pattern observed here for weekdays holds for ordered items generally. If this affective pattern is driven solely by serial position, then associations with early items in *any* sequence should be low in pleasure, low in arousal, and high in dominance, whereas associations with late items should be high in pleasure, high in arousal, and low in dominance. Our own view is that there is more to the days of the week than their order, and that the interplay between weekday and cognition extends beyond sequence memory. The acid test will be whether aspects of cognition that are normally considered distinct from sequence memory (e.g. risk tolerance or face perception) vary systematically over the week. If so, the implications could be profound—not only for individual behavior [44–47], but also for psychological measurement.

Another promising line of research would be to investigate the underlying causes of psychological differentiation among weekdays. Our results already suggest an important role for the weekend in regulating psychological effects: First, the Bank Holiday effect in Study 1 implies that the weekend/working week transition is at least as important as absolute position in the seven-day cycle in determining apparent day [30, 31]. Second, the trajectories of the *Pleasure*, *Arousal*, and *Dominance* ratings in Study 3 reversed abruptly over the weekend, after following monotonic trends through the working week. It is not yet clear whether these effects of weekend are driven by changes in sleep patterns (e.g. reduction of sleep deficit), changes in activities (e.g. home versus work environment), or other factors. Studying populations for whom these factors are dissociated (e.g. shift workers or retirees) should help to disentangle these possibilities.

The present findings already advance our understanding of weekday processing in applied settings. We previously found [24] that weekday affects attendance rate for medical appointments. Confusion over the current day seems unlikely to explain this pattern. A confusability account should predict a midweek peak in missed appointments, as midweek days are the most confusable. The observed pattern of missed appointments follows the pattern of emotional responses to weekday cues seen in Study 3. Specifically, attendance was lower on days that elicit emotionally negative associations (e.g. Monday), and higher on days that elicit emotionally positive associations (e.g. Friday). This pattern suggests that medical appointments might be psychologically harder to face on some days of the week than on others. One way to test this possibility would be to compare weekday effects for more aversive and less aversive appointments. If the weekday effect is mediated by psychological resilience, then it may be stronger when an aversive procedure looms [48, 49].

For now, we show that effects of weekday on cognition can be traced to differences between mental representations of weekdays. Our studies also establish several facts relating weekday and cognition. First, mismatches between apparent day and actual day seem to be surprisingly common. Second, the distribution of mismatches across the week is highly systematic, with higher prevalence on midweek days than on other days. Third, mismatches are mainly intrusions from neighboring days, rather than more distant days. Fourth, a holiday Monday induces a one-day lag between apparent day and actual day. Fifth, the current day is easier to retrieve on Monday and Friday than midweek. Sixth, midweek days elicit fewer semantic associations than other weekdays. Seventh, midweek days occur less frequently than other days in webpages and books. Eighth, the emotional tone of weekday associations brightens from Monday to Friday.

We conclude that midweek days are confusable because their mental representations are sparse and similar. Mondays and Fridays are less confusable, because their mental representations are rich and distinctive. Previous studies have shown that natural temporal cycles (days, months, years) have psychological consequences. The present findings demonstrate that socially constructed temporal cycles can also shape our thinking.

## Supporting Information

S1 Data Includes percentages from Fig 1, reaction times required to produce Fig 2, number of associations required to produce Fig 3(a), pleasure, arousal and dominance ratings required to produce Fig 4 and distances from Fig 5. (ZIP)

## References

[1] Eagleman DM (2008) Human time perception and its illusions. Current Opinion in Neurobiology, 18, 131–136. 10.1016/j.conb.2008.06.002 18639634 PMC2866156

[2] Gregory RL (2009) Seeing through illusions. Oxford University Press, USA.

[3] Tse PU, Intriligator J, Rivest J, Cavanagh P (2004) Attention and the subjective expansion of time. Perception & Psychophysics, 66, 1171–1189. 15751474 10.3758/bf03196844

[4] Kanai R, Watanabe M (2006) Visual onset expands subjective time. Perception & Psychophysics, 68, 1113–1123. 17355036 10.3758/bf03193714

[5] Spence C, Parise C (2010) Prior-entry: A review. Consciousness and cognition, 19, 364–379. 10.1016/j.concog.2009.12.001 20056554

[6] Dijk DJ, Duffy JF, Czeisler CA (1992) Circadian and sleep/wake dependent aspects of subjective alertness and cognitive performance. Journal of Sleep Research, 1, 112–117. 10607036 10.1111/j.1365-2869.1992.tb00021.x

[7] Foster RG, Roenneberg T (2008) Human responses to the geophysical daily, annual and lunar cycles. Current Biology, 18, R784–R794. 10.1016/j.cub.2008.07.003 18786384

[8] Maire M, Reichert CF, Schmidt C (2013) Sleep-wake rhythms and cognition. Journal of Cognitive and Behavioral Psychotherapies, 13, 133–170.

[9] Cajochen C, Altanay-Ekici S, Münch M, Frey S, Knoblauch V, Wirz-Justice A (2013) Evidence that the lunar cycle influences human sleep. Current Biology, 23, 1485–1488. 10.1016/j.cub.2013.06.029 23891110

[10] Chakraborty U (2013) Effects of different phases of the lunar month on humans. Biological Rhythm Research, (ahead-of-print), 1–14.

[11] Golder SA, Macy MW (2011) Diurnal and seasonal mood vary with work, sleep, and daylength across diverse cultures. Science, 333, 1878–1881. 10.1126/science.1202775 21960633

[12] Rohan KJ, Roecklein KA, Haaga DA (2009) Biological and psychological mechanisms of seasonal affective disorder: a review and integration. Current Psychiatry Reviews, 5, 37–47.

[13] Colson FH (1926) The Week: An Essay on the Origin and Development of the Seven-day Cycle. Cambridge University Press.

[14] Zerubavel E (1985) The Seven Day Circle: The History and Meaning of the Week. University of Chicago Press.

[15] Areni CS, Burger M (2008) Memories of “bad” days are more biased than memories of “good” days: past Saturdays vary, but past Mondays are always blue. Journal of Applied Social Psychology, 38 (6), 1395–1415.

[16] Cross F (1973) The behavior of stock prices on Fridays and Mondays. Financial analysts journal, 67–69.

[17] Kamara A (1997) New evidence on the Monday seasonal in stock returns. Journal of Business, 63–84.

[18] Pettengill GN (2003) A survey of the Monday effect literature. Quarterly Journal of Business and Economics, 3–27.

[19] Willich SN, Löwel H, Lewis M, Hörmann A, Arntz HR, Keil U (1994) Weekly variation of acute myocardial infarction. Increased Monday risk in the working population. Circulation, 90, 87–93. 8026056 10.1161/01.cir.90.1.87

[20] Maldonado G, Kraus JF (1991) Variation in suicide occurrence by time of day, day of the week, month, and lunar phase. Suicide and Life-Threatening Behavior, 21, 174–187. 1887454

[21] Stone AA, Schneider S, Harter JK (2012) Day-of-week mood patterns in the United States: On the existence of ‘Blue Monday’, ‘Thank God it’s Friday’ and weekend effects. Journal of Positive Psychology, 7, 306–314.

[22] Jaffe JF, Westerfield R, Ma C (1989) A twist on the Monday effect in stock prices: evidence from the US and foreign stock markets. Journal of Banking & Finance, 13, 641–650.

[23] Johnson H, Brock A, Griffiths C, Rooney C (2005) Mortality from suicide and drug-related poisoning by day of the week in England and Wales, 1993–2002. Health statistics quarterly, 27, 13–16. 16138750

[24] Ellis DA, Jenkins R (2012) Weekday affects attendance rate for medical appointments: Large-scale data analysis and implications. PLoS ONE 7(12): e51365 10.1371/journal.pone.0051365 23272102 PMC3521765

[25] Angie AD, Connelly S, Waples EP, Kligyte V (2011) The influence of discrete emotions on judgement and decision-making: A meta-analytic review. Cognition & Emotion, 25, 1393–1422. 21500048 10.1080/02699931.2010.550751

[26] Tversky A (1977) Features of similarity. Psychological Review, 84, 327–352.

[27] Baddeley AD (1968) How does acoustic similarity influence short-term memory? The Quarterly Journal of Experimental Psychology, 20, 249–264. 5683764 10.1080/14640746808400159

[28] Tulving E (1972) Episodic and semantic memory In Tulving E. and Donaldson W. (Eds.), Organization of Memory (pp. 381–402). New York: Academic Press.

[29] Getty DJ, Swets JA, Swets JB, Green DM (1979) On the prediction of confusion matrices from similarity judgments. Perception & Psychophysics, 26, 1–19.

[30] Huttenlocher J, Hedges LV, Prohaska V (1992) Memory for day of the week: A 5+2 day cycle. Journal of Experimental Psychology: General, 121, 313–325. 1402704 10.1037//0096-3445.121.3.313

[31] Neath I, Brown GD (2006) SIMPLE: Further applications of a local distinctiveness model of memory. Psychology of learning and motivation, 46, 201–244.

[32] Huttenlocher J, Hedges LV, Lourenco SF, Crawford LE, Corrigan B (2007) Estimating stimuli from contrasting categories: Truncation due to boundaries. Journal of Experimental Psychology: General, 136, 502–519. 17696696 10.1037/0096-3445.136.3.502

[33] Hurlstone MJ, Hitch GJ, Baddeley AD (2014) Memory for serial order across domains: An overview of the literature and directions for future research. Psychological Bulletin, 140, 339–373. 10.1037/a0034221 24079725

[34] Estes WK (1997) Processes of memory loss, recovery, and distortion. Psychological Review, 104, 148–169. 9009883 10.1037/0033-295x.104.1.148

[35] Brown GD, Neath I, Chater N (2007) A temporal ratio model of memory. Psychological Review, 114, 539–576. 17638496 10.1037/0033-295X.114.3.539

[36] Tulving E, Madigan SA (1970) Memory and verbal learning. Annual Review of Psychology, 21, 437–484.

[37] Bradley MM, Lang PJ (1999) Affective Norms for English Words (ANEW): Stimuli, instruction manual, and affective ratings (Tech. Report C-1). Gainesville: University of Florida, Center for Research in Psychophysiology.

[38] Areni CS, Burger M, Zlatevska N (2011) Factors affecting the extent of Monday blues: Evidence from a meta-analysis. Psychological Reports, 109, 723–733. 22420107 10.2466/13.20.PR0.109.6.723-733

[39] Michel JB, Shen YK, Aiden AP, Veres A, Gray MK, Team TGB, et al (2011) Quantitative analysis of culture using millions of digitized books. Science, 331, 176–182. 10.1126/science.1199644 21163965 PMC3279742

[40] Leech G, Rayson P, Wilson A (2001) Word frequencies in written and spoken English Harlow, England: Longman.

[41] Vigliocco G, Vinson DP, Damian MF, Levelt W (2002) Semantic distance effects on object and action naming. Cognition, 85, B61–B69. 12169413 10.1016/s0010-0277(02)00107-5

[42] Craik FI, Tulving E (1975) Depth of processing and the retention of words in episodic memory. Journal of Experimental Psychology: General, 104, 268–294.

[43] Anderson JR (1983) A spreading activation theory of memory. Journal of verbal learning and verbal behavior, 22, 261–295.

[44] Bower GH (1981) Mood and memory. American Psychologist, 36, 129–148. 7224324 10.1037//0003-066x.36.2.129

[45] Eich E (1995) Searching for mood dependent memory. Psychological Science, 6, 67–75. 10.1111/1467-9280.00249 11273411

[46] Schwarz N (2000) Emotion, cognition, and decision making. Cognition & Emotion, 14, 433–440.

[47] Loewenstein GF, Weber EU, Hsee CK, Welch N (2001) Risk as feelings. Psychological Bulletin, 127, 267 11316014 10.1037/0033-2909.127.2.267

[48] Redelmeier D, Kahneman D (1996) Patients’ memories of painful medical treatments: Real-time and retrospective evaluations of two minimally invasive procedures. Pain, 66, 3–8. 8857625 10.1016/0304-3959(96)02994-6

[49] Redelmeier DA, Katz J, Kahneman D (2003) Memories of colonoscopy: A randomized trial. Pain, 104, 187–194. 12855328 10.1016/s0304-3959(03)00003-4

